# Lower grip strength and dynamic body balance in women with distal radial fractures

**DOI:** 10.1007/s00198-018-04816-4

**Published:** 2019-01-04

**Authors:** K. Fujita, H. Kaburagi, A. Nimura, T. Miyamoto, Y. Wakabayashi, Y. Seki, H. Aoyama, H. Shimura, R. Kato, A. Okawa

**Affiliations:** 1 Department of Orthopaedic and Spinal Surgery, Graduate School of Medical and Dental Sciences, Tokyo Medical and Dental University, Tokyo, Japan; 2 Department of Functional Joint Anatomy, Graduate School of Medical and Dental Sciences, Tokyo Medical and Dental University, Tokyo, Japan; 3 Department of Orthopedic Surgery, Yokohama City Minato Red Cross Hospital, Kanagawa, Japan; 4 Department of Orthopedic Surgery, Suwa Central Hospital, Nagano, Japan; 5 Department of Orthopedic Surgery, JA Toride Medical Center, Ibaraki, Japan; 6 Department of Orthopedic Surgery, Tokyo Bay Urayasu Ichikawa Medical Center, Chiba, Japan; 7 JA Kyosai Research Institute, Tokyo, Japan

**Keywords:** Distal radial fracture, Fall tendency, Grip strength, TUG

## Abstract

**Summary:**

In this case-control study, we concluded that women with distal radial fractures who were surgically treated showed lower grip strength and dynamic body balancing than those of controls. These results suggest that measurements of grip strength and dynamic body balance may be useful screening tools to assess future fracture risk.

**Introduction:**

Patients with distal radial fractures (DRFs) are at risk of future fragility fractures. However, their physical characteristics and tendencies for falls remain unclear. We aimed to compare the physical characteristics of women with and without distal radial fractures.

**Methods:**

We included 128 women with a DRF as their first fragility fracture (fracture group) who underwent surgical treatment. Concurrently, 128 age- and sex-matched participants without a history of fragility fractures were selected as controls (control group). The participants underwent assessments of grip strength and the body balancing ability test. Measurements were taken twice in the fracture group, at 2 weeks and 6 months postoperatively, and once in the control group. The body balancing ability test included the Functional Reach Test, Timed Up and Go test (TUG), 2-Step test (2ST), and Timed Uni-pedal Stance test. The participants also completed questionnaires about their health.

**Results:**

There were no significant differences (*p* > 0.05) in patient characteristics between the groups. The fracture group showed lower grip strength across all age groups. In the DRF group, prolonged TUG time was observed at 2 weeks postoperatively in all age groups and at 6 months in participants aged 55–74 years; the 2ST score was significantly lower in participants aged between 65 and 74 years.

**Conclusions:**

Women with DRF demonstrated lower grip strength and dynamic body balancing ability. Lower grip strength and dynamic body balancing ability were identified as significant risk factors in women with DRF, suggesting that these may be useful screening tools to assess fracture risk.

**Electronic supplementary material:**

The online version of this article (10.1007/s00198-018-04816-4) contains supplementary material, which is available to authorized users.

## Introduction

Postmenopausal women are at risk of fragility fractures of the spine, hip, proximal humerus, and distal radius [1–3]. Of these, distal radial fractures (DRF) are frequently the first fragility fracture. DRF patients are younger than those with fractures at other sites [4]. Therefore, patients with DRF as the primary fragility fracture are at risk for more morbid fractures, such as fractures of the hip or spine [5, 6]. Along with fracture treatment, intensive planning and care to reduce the risk for future fractures are strongly recommended [7, 8].

Although osteoporosis is recognized as a risk factor for fragility fractures [2, 9], it is a small part of the total risk factors for DRF in women [10]. More than half of DRF patients do not meet the diagnostic criteria of osteoporosis [11]. Usually, DRF results from a fall from standing height [12]. As a result, both muscle weakness and/or impaired body balancing ability are considered important contributing factors for these fractures [1].

Sarcopenia, which is an age-related loss of muscle mass, is related to the risk of falls and osteoporotic fractures [13–15]. Patients with sarcopenia are more likely to experience falls and develop fractures than are patients without fractures. Additionally, uni-pedal standing time and dynamic motion score were reported to be lower in DRF patients than in controls without fractures [11, 16]. However, previous reports have included patients with primary and secondary fragility fractures and those who have undergone surgical and non-surgical treatments. Furthermore, most studies had cross-sectional designs and demonstrated decreased body balancing ability in patients who developed DRF at least 6 months before the study [11, 16]. Therefore, the association between impaired balance and fractures has not been well studied.

We aimed to evaluate the physical characteristics of female patients with DRF as the primary fragility fracture who were treated surgically and to compare them with those of women of similar age without a history of a fragility fracture. We focused on this population to accurately evaluate the relationship between physical status and DRF. We hypothesized that DRF patients would demonstrate weaker grip strength and lower body balancing ability compared with age- and sex-matched control patients.

## Materials and methods

This prospective multi-center study was approved by the institutional review board of Tokyo Medical and Dental University. Informed consent was provided by all participants. Postmenopausal women aged > 40 years with DRF as their first fragility fracture following a fall from standing height or less and who underwent surgery in the eight registered hospitals between January 2015 and December 2015 were recruited into the fracture group. Patients with DRF due to traffic or industrial accidents, multi-organ injuries, dementia, a history of any other fragility fracture, and referral to other hospitals; those who refused to join the study; and those currently on glucocorticoid treatment were excluded (Supplementary Fig. 1). Postmenopausal women without a history of fragility fractures who lived near the hospital were recruited as the control group through advertisements via regional media.

### Outcome measures

All participants completed the paper-based questionnaire about lifestyle habits, including tobacco and alcohol use, general health status including current illnesses and current drug treatments, and comprehensive health questions including treatment for compromised balance or osteoporosis. Grip strength (GS) was measured in kilograms (kg) with a Jamar dynamometer (Sammons Preston, IL, USA), with the elbow flexed at 90° and the forearm in neutral rotation [17]. The mean values of three measurements were recorded. Body balancing ability was measured using four tests: Functional Reach Test (FRT) [18], Timed Up and Go test (TUG) [19], 2 Step test (2ST) [20], and Timed Uni-pedal Stance test with eyes open (TUS) [21]. FRT involves a participant reaching as far forward as possible along a measuring scale with one arm extended and the hand clenched into a fist without losing balance. The mean values of the three trials were recorded in centimeters (cm). FRT was considered to measure semi-static balance ability. TUG determines the time taken by a patient to rise from a chair, walk for 3 m, turn around, and return to sit on the chair. Participants were allowed to practice once, and then the maximum time to complete the test was recorded. TUG was a measure of functional performance and dynamic balancing ability that captures transfers, gait, and turning movements. The 2ST score is the value obtained by dividing an individual’s height by the maximum length of a double stride. The mean values of the two trials were recorded. 2ST was considered to measure the ability of walking and balance along with muscle strength. TUS is performed on a flat surface with both eyes open and both hands placed on the waist. Participants stand unassisted on one leg, and the time (in seconds) from when one foot is flexed off the floor to when it touches the ground or when the standing leg or hands leave the waist is recorded. The maximum time was 60 s. It was a measure considered to assess postural steadiness in a static position by a temporal measurement. The standardized instructions of the assessments in video format were provided to relevant staff in all registered hospitals. Bone mineral density (BMD) was assessed at the lumbar spine (L1–L4) using dual-energy X-ray absorptiometry (DXA, the Horizon DXA system or Discovery DXA system (Hologic Inc., Waltham, MA)) and expressed as the T-score (standard deviation from the mean BMD value of young healthy subjects) according to Japanese diagnostic criteria [22].

### Study design

In the fracture group, we assessed GS of the non-fractured side, four body balancing tests, and BMD at 2 weeks and 6 months after the DRF surgery. In the control group, we assessed GS on both sides and four body balancing tests only once (Supplementary Table 1). Routine physiotherapy for wrist fractures was performed in the fracture group.

### Data analysis

We further divided the two groups into four groups on the basis of age: < 54, 55–64, 65–74, and > 75 years, according to a previous report [23]. Descriptive statistics were calculated to determine the participants’ demographics and clinical characteristics. Between-group differences were assessed using Student’s *t* test for continuous variables and the chi-square test for categorical variables. *P* < 0.05 was considered significant. The GS and body balancing test results of the fracture group at 2 weeks and 6 months after the surgery were compared to those of the control group using Student’s *t* test, followed by application of Bonferroni’s correction. *P* < 0.016 was considered significant. The GS and body balancing test results for the fracture group at 2 weeks and 6 months postoperatively were compared using a paired *t* test; *P* < 0.05 was considered significant. Logistic regression analyses were used to analyze the odds ratios (ORs). Body balancing measurements, including FRT, TUG, 2ST, and TUS scores, and GS were converted into categorical variables on the basis of the normal values for the age group or cut-off values for risk of falls in Japanese individuals.

Finally, if a significant independent association between DRF and functional outcomes was observed, receiver operating characteristic (ROC) curves were generated to determine the optimal cutoff points for the DRF according to specificity. With the identified cutoff points, sensitivity, specificity, positive and negative predictive values, likelihood ratio, and area under the curve (AUC) were calculated.

## Results

### Patient demographics and characteristics

After recruitment of patients and controls, 128 female DRF patients completed the survey at 2 weeks and 6 months after the surgery. The mean age (standard deviation [SD]) was 66.9 ± 9.3 years; 128 age-matched volunteer women without a history of fragility fracture completed the survey (mean age, 65.4 ± 9.5 years). Baseline anthropometric characteristics showed no significant differences between the groups. There were also no significant differences between the groups in terms of hand dominance, age at menopause, time since menopause, and life habits (tobacco and alcohol use). Specific comorbidities during the treatment period known to be related to fall risk did not differ significantly, including diabetes mellitus, hypertension, arrhythmia, ophthalmologic diseases, and osteoarthritis. The number of oral medications was significantly higher in the control group than in the fracture group (Table 1). However, no differences were observed between the groups in the use of medications (Supplementary Table 2). Most of the participants were right-hand dominant, and there were no significant differences in the number of fracture sides (Supplementary Table 3).

**Table 1:** Comparison between the study groups

	Control (*N* = 128)	Fracture (*N* = 128)	*P* value
Age^a^ (years)	65.4 ± 9.5	66.9 ± 9.3	0.73
< 55^b^	17 (13%)	17 (13%)	
55-64^b^	37 (29%)	37 (29%)	
65-74^b^	56 (44%)	56 (44%)	
> 74^b^	18 (14%)	18 (14%)	
Height^a^ (cm)	154.9 ± 5.8	154.0 ± 5.9	0.77
Weight^a^ (kg)	57.3 ± 9.0	54.3 ± 9.3	0.91
Body mass index^a^ (kg/m^2^)	23.9 ± 3.3	22.9 ± 4.2	0.97
Hand dominance^c^			0.76
Right	123 (96.1%)	122 (95.3%)	
Left	5 (3.9%)	6 (4.7%)	
Age of menopause^a^ (years)	49.9 ± 3.7	50.3 ± 4.0	0.45
Time from menopause^a^ (years)	14.7 ± 7.2	16.0 ± 8.6	0.33
Tobacco^c^			
Current smoker	3 (2.3%)	3 (2.3%)	1.00
Previous smoker	6 (4.7%)	7 (5.4%)	0.78
Alcohol^c^	29 (23%)	34 (27%)	0.56
Comorbidities			
Diabetes mellitus^c^	8 (6.3%)	5 (3.9%)	0.39
Hypertension^c^	4 (3.2%)	5 (3.9%)	0.73
Arrhythmia^c^	4 (3.2%)	6 (4.7%)	0.52
Eye diseases^c^	19 (15%)	16 (13%)	0.59
Osteoarthritis^c^	16 (13%)	17 (13%)	0.85
Number of oral medication^c^			
0	49 (38%)	71 (56%)	0.002
1–4	74 (58%)	46 (36%)	
≥ 5	5 (3.9%)	10 (7.9%)	

*P* values < 0.05 are considered significant

^a^Values are presented as means and standard deviations. Independent Student’s *t* test was used to compare the groups

^b^Values are presented as the number of patients and percentages

^c^Values are presented as the number of patients and percentages, and chi-squared test was used for analysis between the groups

### Grip strength and body balancing ability

**Fig. 1:**
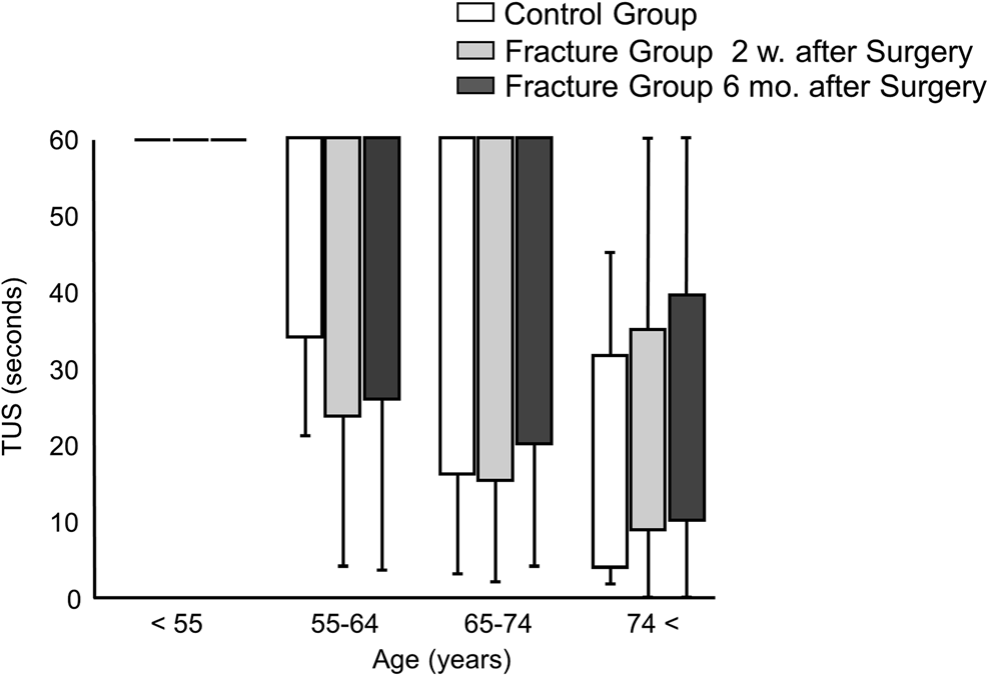
Comparison of the results of TUS between the three groups: control group, fracture group at 2 weeks after surgery, and fracture group at 6 months after surgery. TUS was measured in seconds. Values are plotted as median values and standard deviations. w, weeks; m, months; TUS, Timed Uni-pedal stance test

GS in the fracture group was significantly lower than that in the control group at 2 weeks and 6 months postoperatively in all participants and all age groups (Table 2). Matched control of hand dominance to the fracture group showed the same trend (data not shown). GS in only the dominant hand was significantly higher in the control group than in the fracture group (Supplementary Table 4). Neither fracture side nor hand dominance was significantly different between the groups (Supplementary Table 3). The FRT score demonstrated no significant difference between the groups (Table 3). Within the fracture group, the results at 2 weeks and 6 months postoperatively did not demonstrate a statistical difference. The fracture group demonstrated a significantly higher TUG score at 2 weeks postoperatively than that in the control group in all age groups, and this difference at 6 months postoperatively was observed in patients aged between 55 and 74 years. Additionally, the TUG score demonstrated significant recovery between 2 weeks and 6 months postoperatively in all age groups. The fracture group demonstrated a significantly lower 2ST score in patients aged 65–74 years, and the score significantly recovered between 2 weeks and 6 months postoperatively in all age groups. The TUS score was 60 s (maximum score) among most participants younger than 74 years of age in both groups, so we did not include the TUS score in further analysis (Fig. 1).

**Table 2:** Grip strength in the control and fracture groups

	Control (*N* = 128)		Fracture (*N* = 128)				*P* value		
			2 w. after surgery		6 mo. after surgery		Control to 2 w.	Control to 6 mo.	2 w. to 6 mo.
GS (kg)									
< 55 years	29.5	(25.4 to 33.5)	25.6	(22.7 to 28.4)	25.3	(22.8 to 27.7)	0.010^a^	0.012^a^	0.62^b^
55–64 years	28.1	(26.9 to 29.3)	20.2	(18.1 to 22.4)	21.7	(19.9 to 23.5)	< 0.001^a^	< 0.001^a^	0.005^b^
65–74 years	26.7	(25.8 to 27.6)	19.7	(18.0 to 21.4)	20.7	(19.2 to 22.1)	< 0.001^a^	< 0.001^a^	0.006^b^
> 74 years	23.3	(21.6 to 25.0)	16.3	(14.9 to 17.8)	16.6	(15.2 to 18.0)	< 0.001^a^	< 0.001^a^	0.76^b^

Values are presented as medians and 95% confidence intervals

^a^Student’s *t* test was used for analysis between the groups

^b^Paired sample *t* test was used for analysis between the groups. GS, grip strength; w, weeks; m, months

**Table 3:** Body balancing tests results in the control and fracture groups

	Control (*N* = 128)		Fracture (*N* = 128)				*P* value		
			2 w. after surgery		6 mo. after surgery		Control to 2 w.	Control to 6 mo.	2 w. to 6 mo.
FRT (cm)									
< 55 years	34.6	(32.7 to 36.5)	30.5	(27.7 to 33.3)	34.1	(32.3 to 35.9)	0.03^a^	0.76^a^	0.05^b^
55–64 years	30.9	(29.1 to 32.7)	30.3	(28.1 to 32.5)	32.4	(27.8 to 37.0)	0.68^a^	0.85^a^	0.88^b^
65–74 years	30.1	(28.9 to 31.3)	31.0	(29.4 to 32.5)	31.2	(29.4 to 32.9)	0.37^a^	0.29^a^	0.76^b^
> 74 years	26.3	(23.3 to 29.3)	26.6	(24.5 to 28.7)	27.7	(25.5 to 30.0)	0.87^a^	0.49^a^	0.49^b^
TUG (s)									
< 55 years	5.91	(5.49 to 6.33)	6.66	(6.11 to 7.22)	6.03	(5.69 to 6.36)	0.01^a^	0.67^a^	0.003^b^
55–64 years	5.82	(5.50 to 6.13)	6.90	(6.50 to 7.30)	6.43	(6.14 to 6.73)	< 0.001^a^	0.007^a^	0.007^b^
65–74 years	5.96	(5.76 to 6.16)	7.41	(6.97 to 7.85)	6.79	(6.45 to 7.14)	< 0.001^a^	<0.001^a^	0.002^b^
> 74 years	7.33	(6.68 to 7.98)	9.18	(8.08 to 10.3)	8.17	(7.24 to 9.10)	0.013^a^	0.30^a^	<0.001^b^
2ST									
< 55 years	1.49	(1.36 to 1.61)	1.43	(1.34 to 1.52)	1.54	(1.47 to 1.60)	0.32^a^	0.25^a^	0.008^b^
55–64 years	1.46	(1.41 to 1.51)	1.41	(1.34 to 1.48)	1.50	(1.44 to 1.56)	0.31^a^	0.32^a^	0.02^b^
65–74 years	1.49	(1.46 to 1.53)	1.30	(1.25 to 1.36)	1.42	(1.35 to 1.49)	< 0.001^a^	0.05^a^	<0.001^b^
> 74 years	1.29	(1.20 to 1.39)	1.19	(1.13 to 1.25)	1.28	(1.20 to 1.36)	0.10^a^	0.87^a^	0.04^b^

FRT, Functional Reach test; TUG, Timed Up and Go test; and 2ST, 2 Step test; w, weeks; m, months. FRT, TUG, and 2ST values are presented as medians and 95% confidence intervals

^a^Student’s *t* test was used for analysis between the groups

^b^Paired sample *t* test was used for analysis between the groups

### BMD

In the fracture group, 15% of patients had osteoporosis (T-score < − 2.5), while most of the others had osteopenia (T-score between − 1.0 and − 2.5). The T-scores according to age were as follows: age 54 years and younger, − 0.45 ± 0.98; 55–64 years, − 1.54 ± 1.10; 65–74 years, − 1.95 ± 1.13; and age 75 years and older, − 2.19 ± 1.24.

### ORs of distal radial fractures and cut off values

**Fig. 2:**
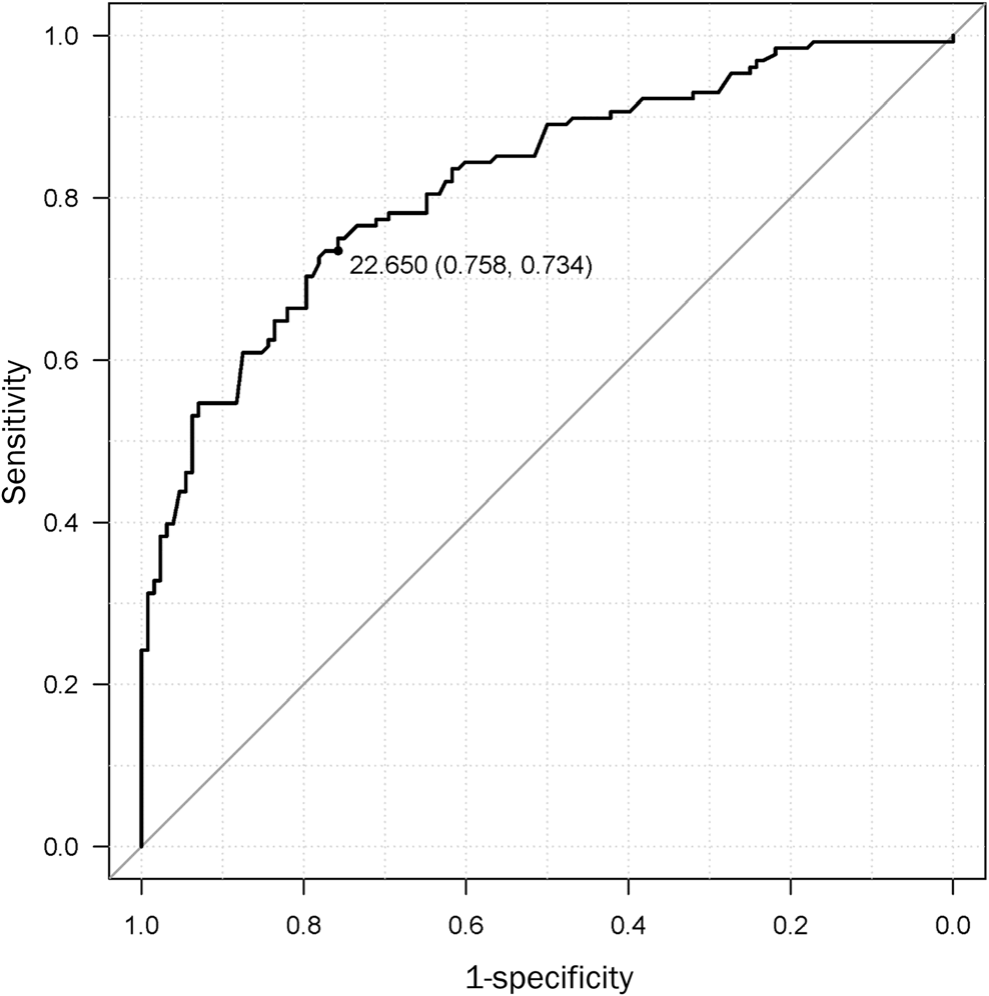
Receiver operating characteristic (ROC) curve for grip strength. Receiver operating characteristic (ROC) curve for grip strength of all participants. The area under the ROC curve (AUC) was 0.821 (95% confidence interval = 0.77–0.872, *P* < 0.0001

We analyzed the association between multiple physical and lifestyle factors with fracture risk using a logistic regression model (Table 4). This analysis demonstrated that alcohol consumption, higher values of TUG, and lower values of 2ST and grip strength were significantly positively correlated with fracture risk. On the other hand, body mass index (BMI) had significant negative correlations with fracture risk. The adjusted OR of a higher TUG score was 8.07 (adjusted 95% confidential interval [CI] 3.18–20.5); that of a lower 2ST score, which reflects lower balancing, was 3.38 (adjusted 95% CI 1.57–7.26); and that of lower GS was 11.5 (adjusted 95% CI 4.98–26.6). Furthermore, ROC curve analyses of the relationship between fracture and GS revealed a cut-off value of fracture risk of 22.7 kg with a sensitivity of 75.8%, specificity of 73.4%, and AUC of 0.821 (Fig. 2).

**Table 4:** Odds ratios, 95% confidential intervals (CI), *P* values, and adjusted values for fracture risk

	Odds ratio	95% CI	*P* value	Adjusted odds ratio	Adjusted 95% CI	Adjusted *P* value
BMI	0.93	(0.87 to 0.99)	0.05	0.88	(0.80 to 0.97)	0.01
Tobacco	6.20	(0.74 to 52.2)	0.47	2.90	(0.19 to 43.6)	0.44
Alcohol	1.23	(0.70 to 2.18)	0.09	3.12	(1.24 to 7.88)	0.02
Oral Medication	0.87	(0.58 to 1.31)	0.49	0.74	(0.49 to 1.12)	0.16
FRT	0.16	(0.08 to 0.30)	< 0.001	0.072	(0.03 to 1.89)	< 0.001
TUG	7.62	(3.82 to 15.2)	< 0.001	8.07	(3.18 to 20.5)	< 0.001
2ST	3.41	(2.03 to 5.71)	< 0.001	3.38	(1.57 to 7.26)	0.002
GS	9.54	(5.13 to 17.7)	< 0.001	11.5	(4.98 to 26.6)	< 0.001

BMI, body mass index; FRT, Functional Reach test; TUG, Timed Up and Go test; 2ST, 2 Step test; and GS, Grip strength

## Discussion

In this study, we observed significant weakness in GS in the fracture group across all age groups at both 2 weeks and 6 months postoperatively. Interestingly, significantly lower TUG scores were observed in the fracture group at 2 weeks postoperatively across all age groups, whereas decreased 2ST scores were observed only in patients aged 65–74 years. On the other hand, we did not observe differences in FRT scores in either group at 2 weeks or 6 months postoperatively. BMD scores in the fracture group were relatively lower; however, a T-score of − 2.5 or lower, which is a criterion to diagnose osteoporosis [24], was observed only in the group aged > 75 years. The other age groups did not meet the diagnostic criteria for osteoporosis but demonstrated signs of osteopenia or low BMD.

Previous reports have shown lower body balancing ability in DRF patients than in healthy patients [25, 26]. However, in those cross-sectional reports, assessments of body balancing ability were performed at least 6 months after fractures [11, 16]. Therefore, it was unclear whether the lower body balance was the cause or effect of DRF. In comparison, in our study, we assessed body balance ability 2 weeks postoperatively, which might be related to the baseline statuses preoperatively, along with the anxiety and fear of falling again. Furthermore, our study included only patients with first fragility fractures to precisely assess body balance ability and future fracture risk in such patients.

In this study, we used four balance tests with low rates of inter- and intra-examiner errors that are easy to perform during routine physical examination [19, 27–30]. While detailed balancing ability studies, such as those that require special tools, are also important, ease of examination was prioritized in this study. Of the four balance tests, FRT has been previously reported to be ineffective in predicting future falls in community-dwelling adults [31]. Our study also did not demonstrate significant differences in FRT scores between the groups, confirming that FRT is not a useful predictor of fractures. Interestingly, we found that TUG and 2ST demonstrated lower values in patients with fractures in most age groups at 2 weeks postoperatively, which, to the best of our knowledge, has not been reported previously.

We also found that lower GS in the fracture group at both 2 weeks and 6 months postoperatively across all ages. Multiple studies have previously reported a close relationship between GS and life span, whole body muscle volume, and physical activity [32–36]; therefore, our results may suggest that GS is a predictor of primary fragility fractures. We believe it is likely that DRF patients had physical decline before the fracture, which then continued after the fracture as well. As we had anticipated, BMD in DRF patients was relatively lower; however, most patients did not meet the criteria for osteoporosis, even though most of them had a low BMD. This observation is in accordance with that of previous reports [10, 37] and implies that physical decline including body balance ability and GS may contribute to the first fragility fracture more than osteoporosis.

Body balancing ability is classified into static and dynamic components [38]. The static component includes TUS and FRT, whereas the dynamic components include TUG and 2ST. Our results demonstrate that DRF patients had lower dynamic body balancing ability, measured by TUG and 2ST. This observation is consistent with the fact that DRF patients are relatively younger and more active than those with spine or hip fractures [39]. Furthermore, dynamic body balancing ability was significantly lower at 2 weeks postoperatively and demonstrated an increasing trend through 6 months. One potential explanation for this observation is that in younger patients, motivation to exercise and prevent future fractures is higher following the fracture. Owing to this bias, we could not clarify the causal relationship clearly between fractures and physical decline in this case-control study. To examine this hypothesis, we plan to assess physical activity levels before and after fractures in a future study. Previous reports that have measured balancing ability at more than 6 months postoperatively have not evaluated fall risk [16].

Several limitations of this study should be acknowledged. First, we focused on patients who underwent surgical correction; therefore, these results are applicable only to patients who were treated surgically. Conservative treatment is usually chosen in patients with severe complications. Therefore, patients who receive conservative treatment may demonstrate greater declines in body balancing ability. Second, we could not assess BMD in the control group. However, the values of BMD in the fracture group were comparable to the Japanese age-matched normative values [40]. BMD could be a potential confounding factor. We intend to conduct a cohort study to assess BMD and physical ability in the future. Third, the BMI of the participants was within the normative range of Japanese women, which is relatively low [41], and this could influence the generalizability of the results.

In summary, we performed a case-control study in women with DRF as the first fragility fracture who were treated surgically and age-matched with women without a history of fragility fractures for assessment of both GS and body balancing ability. Significantly lower GS and dynamic body balancing ability, as measured by TUG and 2ST, were observed in almost all age groups at 2 weeks postoperatively. Lower GS (OR, 11.5), prolonged TUG (OR, 8.07), and lower values of 2ST (OR, 3.38) can be useful and simple approaches in the clinical assessment for predicting the risk of first fragility fracture. Prospective studies with a focus on these assessments and the development of effective exercises to prevent fractures are warranted.

## Electronic supplementary material

Supplementary Figure 1 (DOCX 115 kb)

Supplementary Table 1 (DOCX 15 kb)

Supplementary Table 2 (DOCX 15 kb)

Supplementary Table 3 (DOCX 14 kb)

Supplementary Table 4 (DOCX 14 kb)
