# Supplementary material for: Lower grip strength and dynamic body balance in women with distal radial fractures
Source: Osteoporos Int. 2019 Jan 4;30(5):949–56. doi: 10.1007/s00198-018-04816-4 (PMC6502779; doi:10.1007/s00198-018-04816-4)
Supplement: Supplementary file 1 — (DOCX 115 kb) [file 198_2018_4816_MOESM1_ESM.docx]

**Supplementary figure 1**


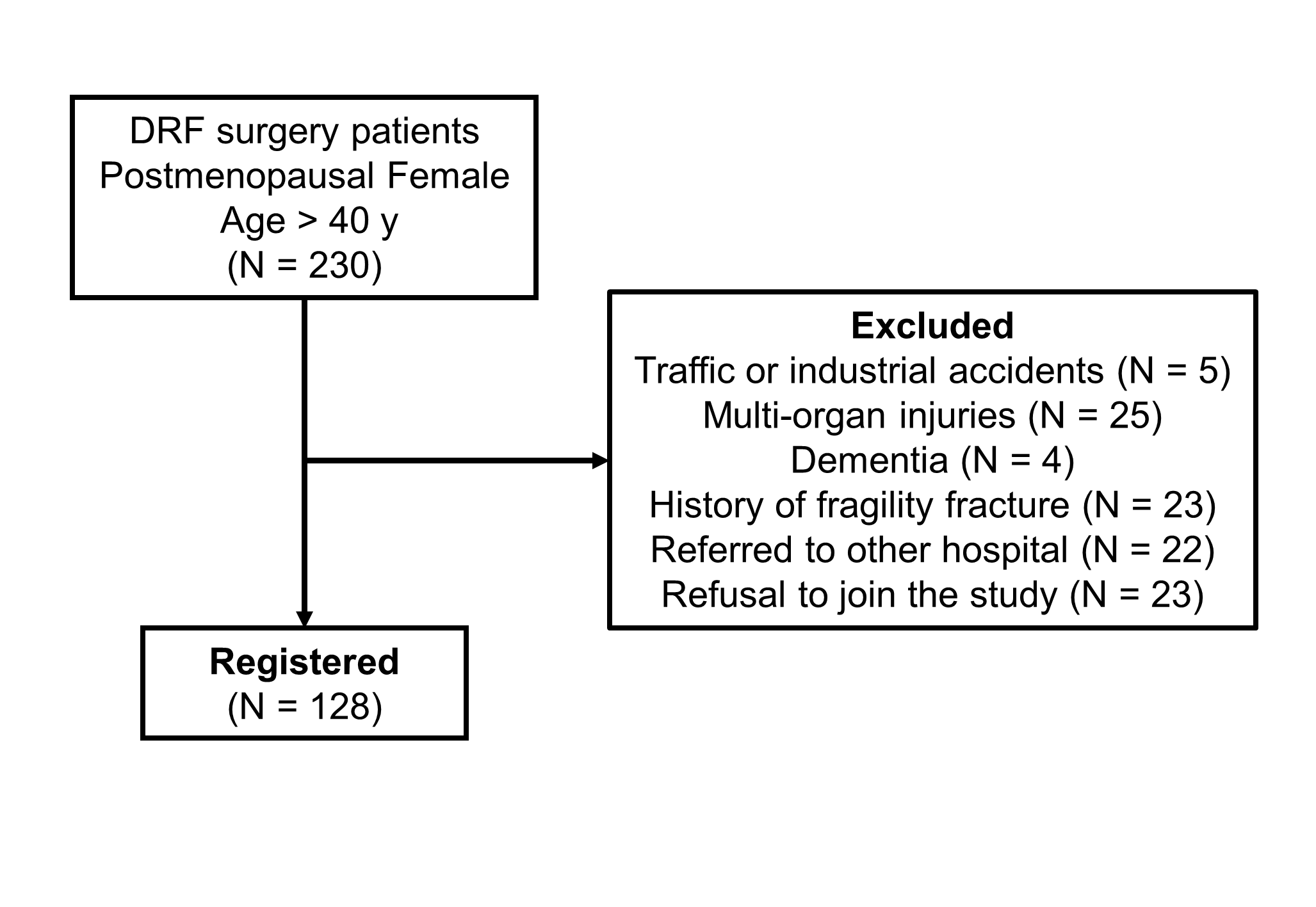


Flowchart of inclusion and exclusion criteria

Postmenopausal women aged >40 years who had a distal radial fracture (DRF) after a fall from standing height or less (N = 230) were included. Patients with DRF due to traffic or industrial accidents (N = 5), and those with multi-organ injuries (N = 25), dementia (N = 4), history of any other fragility fracture (N = 23), referred to other hospital (N = 22), refusal to join the study (N = 23), and those currently on glucocorticoid treatment were excluded.
