# Supplementary material for: Lower grip strength and dynamic body balance in women with distal radial fractures
Source: Osteoporos Int. 2019 Jan 4;30(5):949–56. doi: 10.1007/s00198-018-04816-4 (PMC6502779; doi:10.1007/s00198-018-04816-4)
Supplement: Supplementary file 2 — (DOCX 15 kb) [file 198_2018_4816_MOESM2_ESM.docx]

**Supplementary table 1 Protocol of outcome measurement in the Control and Fracture Groups**

|  | Control (N = 128) | |  | Fracture (N = 128) | | |
| --- | --- | --- | --- | --- | --- | --- |
|  |  |  |  | 2 w. after Surgery |  | 6 mo. after Surgery |
| GS (kg) | ✓(both sides) | |  | ✓(non-fracture side) |  | ✓(non-fracture side) |
| Body balance assessment |  | |  |  |  |  |
| FRT (cm) | ✓ | |  | ✓ |  | ✓ |
| TUG (s) | ✓ | |  | ✓ |  | ✓ |
| 2ST | ✓ | |  | ✓ |  | ✓ |
| TUS (s) | ✓ | |  | ✓ |  | ✓ |
| BMD |  | |  | ✓ |  |  |

GS, Grip strength; FRT, Functional Reach test; TUG, Timed Up and Go test; 2ST, 2 Step test; and BMD, Bone Mineral Density.
