# Supplementary material for: Lower grip strength and dynamic body balance in women with distal radial fractures
Source: Osteoporos Int. 2019 Jan 4;30(5):949–56. doi: 10.1007/s00198-018-04816-4 (PMC6502779; doi:10.1007/s00198-018-04816-4)
Supplement: Supplementary file 3 — (DOCX 15 kb) [file 198_2018_4816_MOESM3_ESM.docx]

**Supplementary table 2 Detailed medication in the Control and Fracture groups**

|  | Control (N = 128) |  | Fracture (N = 128) |  | P value |
| --- | --- | --- | --- | --- | --- |
| Sleep | 8 (6.3%) |  | 2 (1.6%) |  | 0.06 |
| Antihypertension | 27 (21%) |  | 25 (20%) |  | 0.76 |
| Arrhythmia | 3 (2.3%) |  | 3 (2.3%) |  | 1.00 |
| Osteoporosis | 7 (5.5%) |  | 6 (4.7%) |  | 0.78 |
| Diabetes | 6 (4.7%) |  | 5 (3.9%) |  | 0.76 |

Values are presented as the number of patients and percentages, and chi-squared test was used for analysis between the groups.
