# Supplementary material for: Lower grip strength and dynamic body balance in women with distal radial fractures
Source: Osteoporos Int. 2019 Jan 4;30(5):949–56. doi: 10.1007/s00198-018-04816-4 (PMC6502779; doi:10.1007/s00198-018-04816-4)
Supplement: Supplementary file 4 — (DOCX 14 kb) [file 198_2018_4816_MOESM4_ESM.docx]

**Supplementary table 3 Grip strength (kg) of the fracture side and hand dominance in the Fracture Group**

| Fracture (N = 128) | Fracture side | |  |
| --- | --- | --- | --- |
|  | Right (N = 63) | Left (N = 65) | Total |
| Hand Dominance |  |  |  |
| Right (N = 122) | 19.7 (14.3 to 23.7)  (N = 59) | 21.0 (17.3 to 22.8)  (N = 63) | 20.0 (15.8 to 23.0) |
| Left (N = 6) | 14.5*  (N = 4) | 18*  (N = 2) | 14.5 (13.2 to 18.9) |
|  | 19.3 (13.7 to 22.8) | 20.0 (17.3 to 22.5) |  |

Values are presented as medians and 95 % confidence intervals.

^*^ Only median values are presented due to small sample numbers.
