# Supplementary material for: Lower grip strength and dynamic body balance in women with distal radial fractures
Source: Osteoporos Int. 2019 Jan 4;30(5):949–56. doi: 10.1007/s00198-018-04816-4 (PMC6502779; doi:10.1007/s00198-018-04816-4)
Supplement: Supplementary file 5 — (DOCX 14 kb) [file 198_2018_4816_MOESM5_ESM.docx]

**Supplementary table 4 Hand dominance and Grip strength (kg) in the Control and Fracture Groups**

|  | Dominant | non-Dominant | P value |
| --- | --- | --- | --- |
| **GS (kg)** |  |  |  |
| Control (N = 128) | 26.3 (23.3 to 29.2) | 25.7 (22.1 to 28.0) | <0.001 |
| Fracture (N = 128) | 20.0 (16.6 to 22.4) | 19.7 (14.0 to 23.0) | 0.27 |

P values < 0.05 are considered significant.

Values are presented as medians and 95 % confidence intervals.

Student t-test was used for analysis between the groups.
